# Nosocomial candidiasis in Rio de Janeiro State: Distribution and fluconazole susceptibility profile

**DOI:** 10.1590/S1517-838246220120023

**Published:** 2015-06-01

**Authors:** Paulo Murillo Neufeld, Marcia de Souza Carvalho Melhem, Maria Walderez Szeszs, Marcos Dornelas Ribeiro, Efigênia de Lourdes Teixeira Amorim, Manuela da Silva, Marcia dos Santos Lazéra

**Affiliations:** 1 Universidade Federal do Rio de Janeiro, Departamento de Análises Clínicas e Toxicológicas, Universidade Federal do Rio de Janeiro, Rio de Janeiro, RJ, Brasil, Departamento de Análises Clínicas e Toxicológicas, Universidade Federal do Rio de Janeiro, Rio de Janeiro, RJ, Brazil.; 2 Instituto Adolfo Lutz, Serviço de Parasitologia, Instituto Adolfo Lutz, São Paulo, SP, Brasil, Serviço de Parasitologia, Instituto Adolfo Lutz, São Paulo, SP, Brazil.; 3 Serviço de Patologia Clínica, Instituto Estadual de Hematologia Arthur da Siqueira Cavalcante, Rio de Janeiro, RJ, Brasil, Serviço de Patologia Clínica, Instituto Estadual de Hematologia Arthur da Siqueira Cavalcante, Rio de Janeiro, RJ, Brazil.; 4 Setor de Microbiologia e Urinálise, Laboratório Sérgio Franco, Rio de Janeiro, RJ, Brasil, Setor de Microbiologia e Urinálise, Laboratório Sérgio Franco, Rio de Janeiro, RJ, Brazil.; 5 Fundação Oswaldo Cruz, Instituto Nacional de Controle de Qualidade em Saúde, Fundação Oswaldo Cruz, Rio de Janeiro, RJ, Brasil, Instituto Nacional de Controle de Qualidade em Saúde, Fundação Oswaldo Cruz, Rio de Janeiro, RJ, Brazil.; 6 Fundação Oswaldo Cruz, Laboratório de Micologia, Instituto de Pesquisa Clínica Hospital Evandro Chagas, Fundação Oswaldo Cruz, Rio de Janeiro, RJ, Brasil, Laboratório de Micologia, Instituto de Pesquisa Clínica Hospital Evandro Chagas, Fundação Oswaldo Cruz, Rio de Janeiro, RJ, Brazil.

**Keywords:** *Candida*, fluconazole, antifungal susceptibility, disk diffusion method

## Abstract

One hundred and forty-one *Candida* species isolated from clinical specimens of hospitalized patients in Rio de Janeiro, Brazil, during 2002 to 2007, were analized in order to evaluate the distribution and susceptibility of these species to fluconazole. *Candida albicans* was the most frequent species (45.4%), followed by *C. parapsilosis sensu lato* (28.4%), *C. tropicalis* (14.2%), *C. guilliermondii* (6.4%), *C. famata* (2.8%), *C. glabrata* (1.4%), *C. krusei* (0.7%) and *C. lambica* (0.7%). The sources of fungal isolates were blood (47.5%), respiratory tract (17.7%), urinary tract (16.3%), skin and mucous membrane (7.1%), catheter (5.6%), feces (2.1%) and mitral valve tissue (0.7%). The susceptibility test was performed using the methodology of disk-diffusion in agar as recommended in the M44-A2 Document of the Clinical and Laboratory Standards Institute (CLSI). The majority of the clinical isolates (97.2%) was susceptible (S) to fluconazole, although three isolates (2.1%) were susceptible-dose dependent (S-DD) and one of them (0.7%) was resistant (R). The S-DD isolates were *C. albicans*, *C. parapsilosis sensu lato* and *C. tropicalis*. One isolate of *C. krusei* was resistant to fluconazole. This work documents the high susceptibility to fluconazole by *Candida* species isolated in Rio de Janeiro, Brazil.

## Introduction

Since the 1980 decade, invasive fungal infections have grown considerably and the focus of this process has been immunocompromised patients (Beck-Sagué and Jarvis, 1993; Martin *et al.*, 2003). Among the factors that make the defense system fragile and the individuals susceptible to a variety of opportunistic fungi are the seriousness of the base disease, time of permanence in the intensive therapy unit, antibiotic therapy of large spectrum, chemotherapy, radiotherapy, immunosuppressive therapy, central venous catheter, total parenteral feeding, attended ventilation, burns, abdominal surgeries and organ as well as bone marrow transplantations (Colombo and Guimarães, 2003; Colombo *et al.*, 2007; Pemán and Salavert, 2012). Although, new fungal species are described each year as agents of nosocomial infection (Chakrabarti and Singh, 2011; Nucci and Marr, 2005), *Candida* spp. are still considered as the most current pathogen (Alangaden, 2011; Pfaller *et al.*, 2006a). Based on the literature, *C. albicans* is detected in the majority of the cases in Latin America followed by *C. tropicalis*, *C. parapsilosis sensu lato*, and *C. glabrata* (Pfaller *et al.*, 2010). Nevertheless, *C. famata*, *C. kefyr*, *C. guilliermondii*, *C. lusitaniae*, *C. pelliculosa* and *C. rugosa* have presented increasing isolation rates (Colombo *et al.*, 2006; Matta *et al.*, 2007; Pfaller and Diekema, 2007).

Due to the long use for fluconazole to the invasive candidiasis treatment, strains of non-*albicans* species with low susceptibility have appeared in the hospital ambient (Chen *et al.*, 2012). As the resistance to fluconazole can be the cause of therapeutic failure, the fast identification of the etiologic agent and the analysis of the susceptibility profile to the antifungal drugs can help to decide the most appropriate treatment (Montravers and Jabbour, 2006; Pfaller and Diekema, 2007; Shah *et al.*, 2011).

The method employed to evaluate antifungal susceptibility must present a good clinical correlation and has to be reproducible (Hospenthal *et al.*, 2004; Lass-Flörl *et al.*, 2010). Within this context, an effort has been made by the Clinical and Laboratory Standards Institute - CLSI, which developed a reference test to evaluate the *in vitro* susceptibility of *Candida* spp. to fluconazole (CLSI, 2009), using the disk-diffusion in agar methodology (M44-A2). Due to its simplicity, facility of execution and low cost, this method can be easily incorporated in the routine of the public and private clinical laboratories, working as a predictor of clinical response as well as a tool of surveillance and control of the emergence of *Candida* spp. strains with low sensitivity to fluconazole (Pfaller *et al.*, 2004).

The present study describes the distribution of *Candida* species isolated from clinical specimens obtained from hospitalized adult and infant patients in Rio de Janeiro, Brazil, and their susceptibility profile to fluconazole employing the agar disk diffusion method described by CLSI (CLSI, 2009).

## Material and Methods

### Samples

One hundred and forty-one nosocomial isolates of *Candida* spp. (one isolate by patient) were obtained from 2002 to 2007 from five medical centers (a tertiary teaching hospital, a tertiary private hospital, a tertiary militar hospital, a hematological and hemotherapic center and an university pediatric center), one public and one private laboratory of clinical analyses, all of them located in the City of Rio de Janeiro. Despite this localization, the nosocomial samples came from different regions of the State of Rio de Janeiro. In this investigation, the hospital-acquired infections were those that were diagnosed while the patient was hospitalized in the assistance unit. *Candida* species were isolated from blood, catheter, gastrointestinal tract, genitourinary tract, skin lesions and mitral valve tissue from adult and infant immunocompromised patients. The identification of *Candida* isolates and the fluconazole disk diffusion susceptibility testing were performed at the Clinical Mycology Laboratory of Pharmacy College of the Federal University of Rio de Janeiro and at the Mycology Sector of Parasitology Service of Adolfo Lutz Institute, São Paulo, Brazil. All information about the samples were obtained from the data records sent with requests for mycological analyses. However, many of them were incomplete. Thus, it was not possible to classify all samples studied.

### Yeast Identification

All isolates of *Candida* species were identified based on their morphophysiological characteristics. The cultivation in CHROMagar-*Candida* medium (Company, France) was performed to confirm the viability and pureness, as well as for a preliminary identification of the isolates by the production of chromogen pigments (green: *C. albicans*, blue: *C. tropicalis* and rose: *C. krusei*) (Odds and Bernaerts, 1994). The observation of the formation of germinative tube in human serum and the production of chlamydospores in cornmeal agar (Oxoid, England) with tween 80 (Reagen, Brazil) was performed in order to identify *C. albicans* (Dalmau, 1929; Taschdjian *et al.*, 1960). The biochemical identification was conducted through the Vitek commercial system (BioMerieux, France) according to the manufacturer recommendation. The isolates were maintained in sterile distilled water at room temperature up to the moment of the susceptibility tests.

#### Antifungal Susceptibility Testing

The agar disk-diffusion test was performed in accordance to the methodology described in M44-A2 document published by CLSI (CLSI, 2009). Paper disks containing 25 μg of fluconazole (CECON, Brazil) and Petri dishes (90 mm of diameter) containing Mueller-Hinton agar (Difco, England) supplemented with 2% of glycose and 0.5 μg mL^−1^ of methylene blue at a depth of 4.0 mm were used. In order to monitor the precision, accuracy and performance of the test, *Candida albicans* ATCC 90028 and *Candida parapsilosis* ATCC 22019 were used as control strains. Each isolate of *Candida* spp. was subcultivated into plates with Sabouraud dextrose agar (Difco, England), which were incubated at 35–37 °C for 24 h. Five colonies of the isolates were collected and suspended in 5 mL of sterile saline (0.85%).

The turbidity suspension was adjusted to the 0.5 McFarland scale (10^6^ cell/mL) in a spectrophotometer (Biospectro, Brazil) using 530 nm wavelength. The yeast suspension was inoculated using a sterile swab over the surface of agar Mueller-Hinton. The disk with fluconazole was aseptically deposited over the inoculated agar and the plate was incubated aerobically at a 35–37 °C temperature for 24 h. The diameter of the inhibition area was measured for determining the susceptibility and calculating the minimal inhibitory concentration (MIC). The interpretative criteria of the fluconazole disk-diffusion test were those suggested by CLSI 7: susceptible (S): ≥ 19 mm; susceptible-dose dependent (S-DD): 15–18 mm; resistant (R): ≤ 14 mm. The values of the corresponding MIC to these diameters CLSI (CLSI, 2009) are the following: S: MIC ≤ 8 μg mL^−1^; S-DD: MIC 16–32 μg mL^−1^; R: MIC ≥ 64 μg mL^s;1^. The quality control test was conducted every day during the procedure according to CLSI (CLSI, 2009) and the results obtained were within expected limit for each control strain.

## Results

The distribution of *Candida* species at the seven health institutions involved in this investigation is presented in Table 1. From the total of isolations, 43.3% occurred in the Hemotherapy and Hematology Center. The largest percentage of recovery of *Candida* spp. from blood stream infections (46.3%) was also observed in this same institution. In the present study, *Candida albicans* (Table 1) was the yeast with the largest isolation rate (45.4%), followed by *C. parapsilosis sensu lato* (28.4%), *C. tropicalis* (14.2%), *C. guilliermondii* (6.4%) and *C. famata* (2.8%), besides *C. glabrata* (1.4%), *C. krusei* (0.7%) and *C. lambica* (0.7%). The isolates of *Candida* here evaluated were obtained from hospitalized patients with different risk factors, among them, onco-hematological diseases, solid tumors and AIDS.

**Table 1:** Distribution of *Candida* clinical isolates by health centers.

Center	Number of isolates (%)								Total [No. (%)]
		
	*C. albicans*	*C. parapsilosis [sensu lato]*	*C. tropicalis*	*C. guilliermondii*	*C. famata*	*C. glabrata*	*C. krusei*	*C. lambica*	
Tertiary teaching hospital	03 (60)	01 (20)	-	-	-	01 (20)	-		05 (3.5)
Terctiary private hospital	09 (81.8)	01 (9.1)	-	-	-	-	-	01 (9.1)	11 (7.8)
Tertiary militar hospital	02 (100)	-	-	-	-	-	-	-	02 (1.4)
Hematological and hemotherapic center	18 (29.5)	27 (44.3)	05 (8.2)	07 (11.5)	04 (6.5)	-	-	-	61 (43.3)
Pediatric university center	07 (41.2)	06 (35.3)	03 (17.6)	01 (5.9)	-	-	-	-	17 (12.1)
Public clinical laboratory	09 (56.2)	2 (12.5)	03 (18.7)	01 (6.3)	-	01 (6.3)	-	-	16 (11.3)
Private clinical laboratory	16 (55.2)	03 (10.3)	09 (31)	-	-	-	1 (3.5)	-	29 (20.6)
Overall	64 (45.4)	40 (28.4)	20 (14.2)	09 (6.4)	04 (2.8)	02 (1.4)	01 (0.7)	01 (0.7)	141 (100)

Blood (47.5%), respiratory tract (17.7%), urinary tract (16.3%), skin and mucous membrane (7.1%), catheter (5.6%), feces (2.1%) and biopsy of the mitral valve (0.7%) were the sources of isolation. It was not possible to define anatomical localization for 3.0% of the isolates. The distribution of *Candida* isolates by clinical specimens is summarized in the Table 2. *C. albicans* and *C. parapsilosis sensu lato* were the most frequent species isolates from hemoculture, presenting rates of 41.8% and 37.3%, respectively. Other yeasts isolated from hemoculture were *C. tropicalis* (10.4%), *C. guilliermondii* (6.0%), *C. famata* (3.0%) and *C. glabrata* (1.5%). From samples of catheter only *C. albicans* (62.5%) and *C. parapsilosis sensu lato* (37.5%) were isolated. The single isolate of *C. krusei* was retrieved from respiratory specimens.

**Table 2:** Distribution of *Candida* isolates by clinical specimens.

Species	Number of isolates (%)								Total [No. (%)]
		
	Blood	Catheter	Urinay tract	Respiratory tract	Skin/soft tissue	Stool	Mitral valve	NC	
*C. albicans*	28 (41.8)	05 (62.5)	13 (56.6)	12 (48)	03 (30)	02 (66.7)	01 (100)	-	64 (45.4)
*C. parapsilosis [sensu lato]*	25 (37.3)	03 (37.5)	04 (17.4)	03 (12)	05 (50)	-	-	-	40 (28.4)
*C. tropicalis*	07 (10.4)	-	02 (8.7)	07 (28)	01 (10)	-	-	03 (75)	20 (14.2)
*C. guilliermondii*	04 (6)	-	03 (13)	01 (4)	-	01 (33.3)	-	-	09 (6.4)
*C. famata*	02 (3)	-	01 (4.3)	-	-	-	-	01 (25)	04 (2.8)
*C. glabrata*	01 (1.5)	-	-	-	01 (10)	-	-	-	02 (1.4)
*C. krusei*	-	-	-	01 (4)	-	-	-	-	01 (0.7)
*C. lambica*	-	-	-	01 (4)	-	-	-	-	01 (0.7)
Total	67 (47.5)	08 (5.6)	23 (16.3)	25 (17.7)	10 (7.1)	03 (2.1)	01 (0.7)	04 (3)	141 (100)

NC, not-classified specimens.

The diameters of inhibition zones produced by the agar disk-diffusion for all of the isolates tested varied from 10 to 50 mm with average value of 35.8 mm. Table 3 presents the intervals and the means of the diameters from the inhibition zone for *Candida* species regarding the relationship to different anatomical sites. Among the species, *C. krusei* (10 mm) presented the smallest inhibition diameter. *C. albicans* (38.6 mm), *C. parapsilosis sensu lato* (36.5 mm) and *C. guilliermondii* (33 mm) were the species that produced diameters with higher average values. Based on diameters size of the inhibition zone, the general profile of susceptibility to fluconazole was the following: 0.7% of the isolates were resistant (MIC ≥ 64 μg mL^−1^), 2.1% were susceptible-dose dependent (MIC 16–32 μg mL^−1^) and 97.2% were susceptible (MIC ≤ 8μg mL^−1^). Table 4 shows the susceptibility to fluconazole separately by species. The resistance to fluconazole was a phenomenon restricted to the only isolate of *C. krusei* in the present investigation. *C. albicans*, *C. parapsilosis sensu lato* and *C. tropicalis*, although clearly susceptible, also were susceptible-dose dependent isolates. The other species, *C. famata*, *C. glabrata*, *C. guilliermondii* and *C. lambica* presented susceptibility rates of 100%.

**Table 3:** Range and average zone diameters (mm) by *Candida* isolates and specimen types.

Specimens	Range (average) [mm]								Total range (average)

	*C. albicans*	*C. parapsilosis* [*sensu lato*]	*C. tropicalis*	*C. guilliermondii*	*C. famata*	*C. glabrata*	*C. krusei*	*C. lambica*	
Blood	16–40 (40.2)	15–50 (37.2)	26–37 (30.5)	28–36 (32.5)	30–32 (31)	35	-	-	15–50 (37.2)
Catheter	30–48 (40.8)	22–44 (36.6)	-	-	-	-	-	-	22–48 (39.2)
Urinary tract	30–44 (39.9)	30–38 (34.7)	30–33 (31.5)	26–38 (32)	34	-	-	-	26–44 (34.7)
Repiratory tract	30–47 (37)	30–36 (33.2)	30–36 (33.6)	20	-	-	10	24	10–47 (33)
Skin/soft tissue	32–50 (38.3)	30–48 (37.2)	38	-	-	20	-	-	20–50 (35.9)
Stool	32–46 (39)	-	-	33	-	-	-	-	32–46 (37)
Mitral valve	37	-	-	-	-	-	-	-	37
NC	-	-	15–35 (26)	-	25	-	-	-	15–35 (25.7)
Total	16–50 (38.6)	15–50 (36.5)	15–38 (31.2)	20–38 (33)	25–34 (30.2)	20–35 (27.5)	10	24	10–50 (35.8)

NC, not-classified specimens.

**Table 4:** Fluconazole susceptibility of 141 *Candida* clinical isolates.

Species	Clinical specimens	Fluconazole susceptibility category (%)		

		S	S-DD	R
*C. albicans*	Blood	96.4	3.6	-
	Non-blood	100	-	-
	All	98.4	1.6	-
*C. parapsilosis [sensu lato]*	Blood	96	4.0	-
	Non-blood	100	-	-
	All	97.5	2.5	-
*C. tropicalis*	Blood	100	-	-
	Non-blood	100	-	-
	NC	66.7	33.3	-
	All	95	5	-
*C. guilliermondii*	Blood	100	-	-
	Non-blood	100	-	-
	All	100	-	-
*C. famata*	Blood	100	-	-
	Non-blood	100	-	-
	NC	-	-	-
	All	100	-	-
*C. glabrata*	Blood	100	-	-
	Non-blood	100	-	-
	All	100	-	-
*C. krusei*	Blood	-	-	-
	Non-blood	-	-	100
	All	-	-	100
*C. lambica*	Blood	-	-	-
	Non-blood	100	-	-
	All	100	-	-
Overall		97.2	2.1	0.7

S, susceptible; S-DD, susceptible-dose dependent; R, resistant; NC, not-classified specimens.

## Discussion

From the medical centers analyzed, the one specialized in the treatment of hematological patients was the center that presented the largest rate of fungal isolation, including isolates from hemocultures (46.3%). Because of its consumptive nature and its more aggressive treatment protocols, the hematological pathologies, in general, make the patients extremely susceptible to invasive fungal infections, therefore becoming one of the major risk factors (Wisplinghoff *et al.*, 2003b). High percentages of fungal recovery from these institutions (or hematological unit) are to be expected (Martin *et al.*, 2003). Within the group of tertiary hospitals and the Pediatric Care Center, the percentages of positive hemoculture were 6.0% and 13.4%, respectively. Similar rates have been documented by different authors (Velasco and Bigni, 2008; Velasco *et al.*, 2000; Wisplinghoff *et al.*, 2003a). Based on these isolations, nowadays the genus *Candida* is considered the third more frequent pathogen in infections of stream blood, after *Staphylococcus epidermidis* and *S. aureus* (Wisplinghoff *et al.*, 2003b, 2004).

The results here documented highlight the high incidence of *C. albicans* in hospitalized patients. In the present work, *C. albicans* represented 41.8% of all isolates from the hemoculture. This percentage agrees with the data recently pointed out regarding this species (48.7%) by the International Program of Epidemiologic Surveillance (SENTRY), which investigated the distribution of *Candida* species isolated from candidemias and their susceptibility profile to antifungics in North America, Latin America and Europe (Messer *et al.*, 2006). In Brazil, *C. albicans* has been equally the most isolated yeast in candidemias in many regions of the country (Aquino *et al.*, 2005; Barberino *et al.*, 2006; Passos *et al.*, 2007). The explanation for the fact that *C. albicans* presents the highest percentage of recovery may be related to its large adaptability and pathogenic versatility (Kumamoto and Vinces, 2005).

In spite of *C. albicans* being the most frequent agent in this investigation (41.8%), as a whole, the non-*C. albicans Candida* species represented the majority of the isolates (58,2%) with preponderancy of *C. parapsilosis sensu lato* (37.3%) and *C. tropicalis* (10.4%). Currently, *C. parapsilosis sensu lato* and *C. tropicalis* correspond conjunctly to about 70% of the non-*C. albicans Candida* isolates from Brazilian candidemias (Colombo *et al.*, 2006). The progressive increase in the rates of recovery of non-*C. albicans Candida* species has been widely related (Bassetti *et al.*, 2011; Pfaller *et al.*, 2010). In Brazil, this tendency was equally confirmed (Nucci *et al.*, 2010; Sampaio-Camargo *et al.*, 2010) and isolation rates up to 75% have been reported to non-*C. albicans Candida* species (Pasqualotto *et al.*, 2005). The emergence of non-*C. albicans Candida* isolates may be due to the selection of more resistant strains because of ostensive use of azoles derivatives (Mario *et al.*, 2012).

In this investigation, blood was the main source of *Candida* species (47.5%). Nevertheless, the respiratory tract (17.7%), urinary tract (16.3%), skin and mucous (7.1%) also contributed as important sources. In accordance to the findings of Comert *et al.* (2006), *C. parapsilosis sensu lato*, *C. tropicalis* and *C guilliermondii* in the present study were also the main non-*C. albicans Candida* species recovered from non-sterile specimens. In spite of the hemoculture being the main marker of invasive infection (Martin *et al.*, 2003) and the non-sterile specimens being considered of relative importance to the infection diagnostic (Wang *et al.*, 2004), the isolation of *Candida* spp. from these specimens may present certain predictive value for candidemias (Sandford *et al.*, 1980), because many invasive processes are associated to the host's microbiota (Agvald-Öhman *et al.*, 2007).

The invasive infections represent high cost to human economy. In the United States the annual cost with these infections is about US$ 17 billions (Martin *et al.*, 2003). For the candidemia treatment, a cost of about US$1 billion per year is estimated (Pfaller *et al.*, 2006b). Among the different factors that contribute in a critical way to calculate economical and social costs of candidemias, it is the microbial resistance to the antifungics of clinical use (Pfaller *et al.*, 2006b, 2007). As a result, the demand for susceptibility tests has been growing (Pfaller, 2012). The agar disk diffusion method may be useful for the selection of more effective, non-toxic and less expensive therapies (Pfaller *et al.*, 2007; Shah *et al.*, 2011). Its effectiveness in the confirmation of the susceptibility or in the detection of resistance to fluconazole has been evaluated in various multicentric international studies (Pfaller *et al.*, 2005, 2007). Good levels of agreement (87.4 to 97%) between zone diameters by disk-diffusion method and MIC by CLSI reference microdilution method has been found (Barry *et al.*, 2002; Rodero *et al.*, 2006), suggesting an *in vivo/in vitro* correlation equivalent to the reference method (Meis *et al.*, 2000).

In the present study it was demonstrated the low percentage (0.7%) of resistance to fluconazole, corroborating with what has been reported previously by other authors (Azevedo, 2011; Pfaller *et al.*, 2003, 2004). The only resistant isolate was *C. krusei* that was recovered from the respiratory tract. The resistance of this yeast was expected, since it is considered intrinsically resistant to fluconazole (Comert *et al.*, 2006). In spite of the occurrence of susceptibility-dose dependent (2.1% from the total of isolates), 98.4% of *C. albicans* isolates, 97.5% of *C. parapsilosis sensu lato* isolates and 95.0% of *C. tropicalis* isolates were susceptible to fluconazole. Pfaller *et al.* (2005, 2007) also reported similar rates from global studies, which included the Latin America and Brazil. To *C. famata*, *C. glabrata*, *C. guilliermondii* and *C. lambica*, the fluconazole was 100% effective. Nevertheless, acquired resistance within isolates of *C. glabrata* has been reported (Colombo *et al.*, 2006; Matta *et al.*, 2007). The high susceptibility of *Candida* isolates to fluconazole described here is also very relevant, since this antifungal medicine is the main available azole drug to treat invasive fungal infections within Brazilian public hospitals (Brasil, 2007). The simplicity and validity of the use of the disk-diffusion method were also confirmed.

The study on resistance to fluconazole and other antifungals comparing CLSI disk-diffusion method to CLSI microdilution method and to EUCAST method was conducted and the results will be published soon, as well as the results of the study comparing the genotypic and phenotypic identification of these isolates.

## References

[B01] Agvald-Öhman C, Klingspor L, Hjelmqvist H (2007). Invasive candidiasis in long-term patients at a multidisciplinary
intensive care unit: Candida colonization index, risk factors, treatment and
outcome. Scand J Infect Dis.

[B02] Alangaden GJ (2011). Nosocomial fungal infections: epidemiology, infection control,
and prevention. Infect Dis Clin North Am.

[B03] Aquino VR, Lunardi LW, Goldani LZ (2005). Prevalence, susceptibility profile for fluconazole and risk
factors for candidemia in a tertiary care hospital in southern
Brazil. Braz J Infect Dis.

[B04] Azevedo AC, Bizerra FC, da Matta DA (2011). *In vitro* susceptibility of a large collection of
*Candida* Strains against fluconazole and voriconazole by
using the CLSI disk diffusion assay. Mycopathologia.

[B05] Barberino MG, Silva N, Rebouças C (2006). Evaluation of blood stream infections by *Candida*
in three tertiary hospitals in Salvador, Brazil: a case-control
study. Braz J Infect Dis.

[B06] Barry AL, Pfaller MA, Rennie RP (2002). Precision and accuracy of fluconazole susceptibility tests by
broth microdilution, Etest and disk diffusion methods. Antimicrob Agents Chemother.

[B07] Bassetti M, Taramasso L, Nicco E (2011). Epidemiology, species distribution, antifungal susceptibility and
outcome of nosocomial candidemia in a tertiary care hospital in
Italy. PLoS One.

[B08] Beck-Sagué CM, Jarvis WR, National Nosocomial Infections Surveillance System (1993). Secular trends in the epidemiology of nosocomial fungal
infections in the United States, 1980–1990. J Infect Dis.

[B09] Brasil, Ministério da Saúde. Secretaria de Ciência, Tecnologia e
Insumos Estratégicos. Departamento de Assistência Farmacêutica e Insumos
Estratégicos (2007). Relação Nacional de Medicamentos Essenciais: RENAME/Ministério da Saúde,
Secretaria de Ciência, Tecnologia e Insumos Estratégicos, Departamento de
Assistência Farmacêutica e Insumos Estratégicos.

[B10] Chakrabarti A, Singh R (2011). The emerging epidemiology of mould infections in developing
countries. Curr Opin Infect Dis.

[B11] Chen TC, Chen YH, Chen YC (2012). Fluconazole exposure rather than clonal spreading is correlated
with the emergence of *Candida glabrata* with
cross-resistance to triazole antifungal agents. Kaohsiung J Med Sci.

[B12] CLSI - Clinical and Laboratory Standards Institute (2009). Publication M44-A2: Method for Antifungal Disk Diffusion Susceptibility
Testing of Yeasts; Approved Guideline - Second Edition.

[B13] Colombo AL, Nucci M, Park BJ (2006). Epidemiology of candidemia in Brazil: a nationwide sentinel
surveillance of candidemia in eleven medical centers. J Clin Microbiol.

[B14] Colombo AL, Guimarães T (2003). Epidemiologia das infecções hematogênicas por
*Candida* spp. Rev Soc Br Med Trop.

[B15] Colombo AL, Guimarães T, Silva LR (2007). Prospective observational study of candidemia in São Paulo,
Brazil: incidence rate, epidemiology, and predictors of
mortality. Infect Control Hosp Epidemiol.

[B16] Comert F, Kulah C, Aktas E (2006). Identification of *Candida* species isolated from
patient in intensive care unit and *in vitro* susceptibility
to fluconazole for a 3-year period. Mycoses.

[B17] Dalmau LM (1929). Observations on mycological technique with particular references
to pathogenic fungi. Porto Rico J Pub Heath Trop Med.

[B18] Hospenthal DR, Murray CK, Rinaldi MG (2004). The role of antifungal susceptibility testing in the therapy of
candidiasis. Diagn Microbiol Infect Dis.

[B19] Kumamoto CA, Vinces MD (2005). Contributions of hyphae and hypha-co-regulated genes to
*Candida albicans* virulence. Cell Microbiol.

[B20] Lass-Flörl C, Perkhofer S, Mayr A (2010). In vitro susceptibility testing in fungi: a global perspective on
a variety of methods. Mycoses.

[B21] Mario DA, Denardi LB, Bandeira LA (2012). The activity of echinocandins, amphotericin B and voriconazole
against fluconazole-susceptible and fluconazole-resistant Brazilian
*Candida glabrata* isolates. Mem Inst Oswaldo Cruz.

[B22] Martin GS, Mannino DM, Eaton S (2003). The Epidemiology of sepsis in the United States from 1979 through
2000. N Engl J Med.

[B23] Matta DA, Almeida LP, Machado AM (2007). Antifungal susceptibility of 1000 *Candida*
bloodstream isolates to 5 antifungal drugs: Results of a multicenter study
conducted in São Paulo, Brazil, 1995–2003. Diagn Microbiol Infect Dis.

[B24] Meis J, Petrou M, Bille J (2000). A global evaluation of the susceptibility of Candida species to
fluconazole by disk diffusion. Global Antifungal Surveillance
Group. Diagn Microbiol Infect Dis.

[B25] Messer SA, Jones RN, Fritsche TR (2006). International surveillance of *Candida* spp. and
*Aspergillus* spp.: report from the SENTRY Antimicrobial
Surveillance Program (2003). J Clin Microbiol.

[B26] Montravers P, Jabbour K (2006). Clinical consequences of resistant *Candida*
infections in intensive care. Int J Antimicrob Agents.

[B27] Nucci M, Queiroz-Telles F, Tobón AM (2010). Epidemiology of opportunistic fungal infections in Latin
America. Clin Infect Dis.

[B28] Nucci M, Marr KA (2005). Emerging fungal diseases. Clin Infect Dis.

[B29] Odds FC, Bernaerts R (1994). CHROMagar *Candida*, a new differential isolation
medium for presumptive identification of clinically important
*Candida* species. J Clin Microbiol.

[B30] Pasqualotto AC, Nedel WL, Machado TS (2005). Risk factors and outcome for nosocomial breakthrough
candidaemia. J Infec.

[B31] Passos XS, Costa CR, Araújo CR (2007). Species distribution and antifungal susceptibility patterns of
*Candida* spp. bloodstream isolates from a Brazilian
tertiary care hospital. Mycopathologia.

[B32] Pemán J, Salavert M (2012). Epidemiología general de la enfermedad fúngica
invasora. Enferm Infecc Microbiol Clin.

[B33] Pfaller MA (2012). Antifungal drug resistance: mechanisms, epidemiology, and
consequences for treatment. Am J Med.

[B34] Pfaller MA, Boyken L, Hollis RJ (2006a). *In vitro* susceptibilities of
*Candida* spp. to caspofungin: four years of global
surveillance. J Clin Microbiol.

[B35] Pfaller MA, Boyken L, Messer SA (2005). Comparison of results of voriconazole disk diffusion testing for
*Candida* species with results from a central reference
laboratory in the ARTEMIS global antifungal surveillance
program. J Clin Microbiol.

[B36] Pfaller MA, Diekema DJ (2007). Epidemiology of invasive candidiasis: a persistent public health
problem. Clin Microbiol Rev.

[B37] Pfaller MA, Diekema DJ, Gibbs DL (2010). Results from the ARTEMIS disk global antifungal Surveillance
study, 1997 to 2007: a 10.5 year analysis of susceptibilities of
*Candida* species to fluconazole and voriconazole as
determined by CLSI standardized disk diffusion. J Clin Microbiol.

[B38] Pfaller MA, Diekema DJ, Gibbs DL (2007). Results from the ARTEMIS disk global antifungal surveillance
study, 1997 to 2005: an 8.5-year analysis of susceptibilities of
*Candida* species and other yeast species to fluconazole
and voriconazole determined by CLSI standardized disk diffusion
testing. J Clin Microbiol.

[B39] Pfaller MA, Diekema DJ, Messer SA (2003). Activities of fluconazole and voriconazole against 1.586 recent
clinical isolates of *Candida* species determined by broth
microdilution disk diffusion and e-test methods: report from the ARTEMIS
global antifungal susceptibility program 2001. J Clin Microbiol.

[B40] Pfaller MA, Diekema DJ, Sheehan DJ (2006b). Interpretive breakpoints for fluconazole and
*Candida* revisited: a blueprint for the future of
antifungal susceptibility testing. Clin Microbiol Rev.

[B41] Pfaller MA, Hazen KC, Messer SA (2004). Comparison of results of fluconazole disk diffusion testing for
*Candida* species with results from a central reference
laboratory in the ARTEMIS global antifungal surveillance
program. J Clin Microbiol.

[B42] Rodero L, Córdoba S, Vivot W (2006). Método de difusión con discos para la determinación de
sensibilidad a fluconazol en aislamientos de *Candida*
spp. Rev Argent Microbiol.

[B43] Sampaio-Camargo TZ, Marra AR, Silva CV (2010). Secular trends of candidemia in a tertiary care
hospital. Am J Infect Control.

[B44] Sandford GR, Merz WG, Wingard JR (1980). The value of fungal surveillance cultures as predictors of
sistemic fungal infections. J Infect Dis.

[B45] Shah DN, Yau R, Weston J (2011). Evaluation of antifungal therapy in patients with candidaemia
based on susceptibility testing results: implications for antimicrobial
stewardship programmes. J Antimicrob Chemother.

[B46] Taschdjian CL, Burchall JJ, kozinn PJ (1960). Rappid identification of *Candida albicans* by
filamentation on serun and serun substitutes. Am J Dis Children.

[B47] Velasco E, Bigni R (2008). A prospective cohort study evaluating the prognostic impact of
clinical characteristics and comorbid conditions of hospitalized adult and
pediatric cancer patients with candidemia. Eur J Clin Microbiol Infect Dis.

[B48] Velasco E, Thuler LC, Martins CA (2000). Epidemiology of bloodstream infections at a cancer
center. São Paulo Med J.

[B49] Wang JL, Chang SC, Hsueh PR (2004). Species distribution and fluconazole susceptibility of
*Candida* clinical isolates in a medical center in
2002. J Microbiol Immunol Infect.

[B50] Wisplinghoff H, Bischoff T, Tallent SM (2004). Nosocomial bloodstream infections in US hospitals: analysis of
24,179 cases from a prospective nationwide surveillance
study. Clin Infect Dis.

[B51] Wisplinghoff H, Seifert H, Tallent SM (2003a). Nosocomial bloodstream infections in pediatric patients in United
States hospitals: epidemiology, clinical features and
susceptibilities. Pediatr Infect Dis J.

[B52] Wisplinghoff H, Seifert H, Wenzel RP (2003b). Current trends in the epidemiology of nosocomial bloodstream
infections in patients with hematological malignancies and solid neoplasms
in hospitals in the United States. Clin Infect Dis.

